# A case of thyroid metastasis from pancreatic cancer: case report and literature review

**DOI:** 10.1186/1472-6823-14-6

**Published:** 2014-01-15

**Authors:** Alessandro P Delitala, Gianpaolo Vidili, Alessandra Manca, Upinder Dial, Giuseppe Delitala, Giuseppe Fanciulli

**Affiliations:** 1 Department of Biomedical Science, University of Sassari, Sassari, Viale San Pietro 8, 07100 Sassari, Italy; 2 Department of Clinical and Experimental Medicine, University of Sassari, Azienda Ospedaliera Universitaria, Sassari, Italy; 3 Department of Pathology, University of Sassari, Azienda Ospedaliera Universitaria, Sassari, Italy

**Keywords:** Contrast enhanced ultrasonography, Core-biopsy, Pancreatic adenocarcinoma, Thyroid metastasis

## Abstract

**Background:**

Thyroid metastases are clinically rare, and usually occur in patients with a history of prior malignancy and when there are metastases elsewhere. Metastases of pancreatic carcinoma to the thyroid are extremely rare, with only three cases reported in the literature.

**Case presentation:**

We report a patient who had a pancreatic carcinoma with metastasis to the thyroid as initial clinical presentation of the disease. A 63-year-old man with a history of weight loss and fatigue presented with cervical lymphadenopathies and a large nodule in the right lobe of the thyroid. A fine needle aspiration of the nodule gave inconclusive cytological results for the origin of the neoplastic cells. An ultrasound-guided core biopsy revealed the presence of a poorly differentiated adenocarcinoma infiltrating the thyroid with atrophic thyroid follicles. Immunohistochemical staining of the lesion was strongly positive for Cytokeratin 19 suggesting a pancreatic origin of the metastasis. A contrast CT scan demonstrated an enlargement of the pancreatic body, dilatation of the pancreatic duct, diffuse retroperitoneal, paraaortic and cervical lymphadenopathy and secondary lesions in the liver.

**Conclusion:**

Metastases to the thyroid from pancreatic carcinoma are extremely rare. A core biopsy of the lesion excluded a thyroid carcinoma and permitted the diagnosis of the primary neoplasm.

## Background

The thyroid gland is a known but unusual site for metastatic tumours from various primary sites. Although clinically apparent metastases from nonthyroid malignancies (NTMs) to the thyroid gland are uncommon, metastases to the thyroid have been reported in 1.4-3.0% of all patients who had surgery for thyroid malignancy [1,2]. Moreover, a wide range of prevalence, from 1.9% to 24%, has been reported from autopsy series [3,4]. The most common NTMs that metastasize to the thyroid are kidney, colorectal, lung and breast carcinomas.

Metastasis of pancreas carcinoma to the thyroid is extremely rare and only three cases have been previously reported. We present a case of thyroid metastasis from a pancreatic carcinoma in which the metastasis was the initial clinical presentation.

## Case presentation

We present the case of a Caucasian 63-year-old man who had an 8 –week history of 10 kilogram weight loss, profound fatigue and cervical lymphadenopathy.

**Figure 1:**
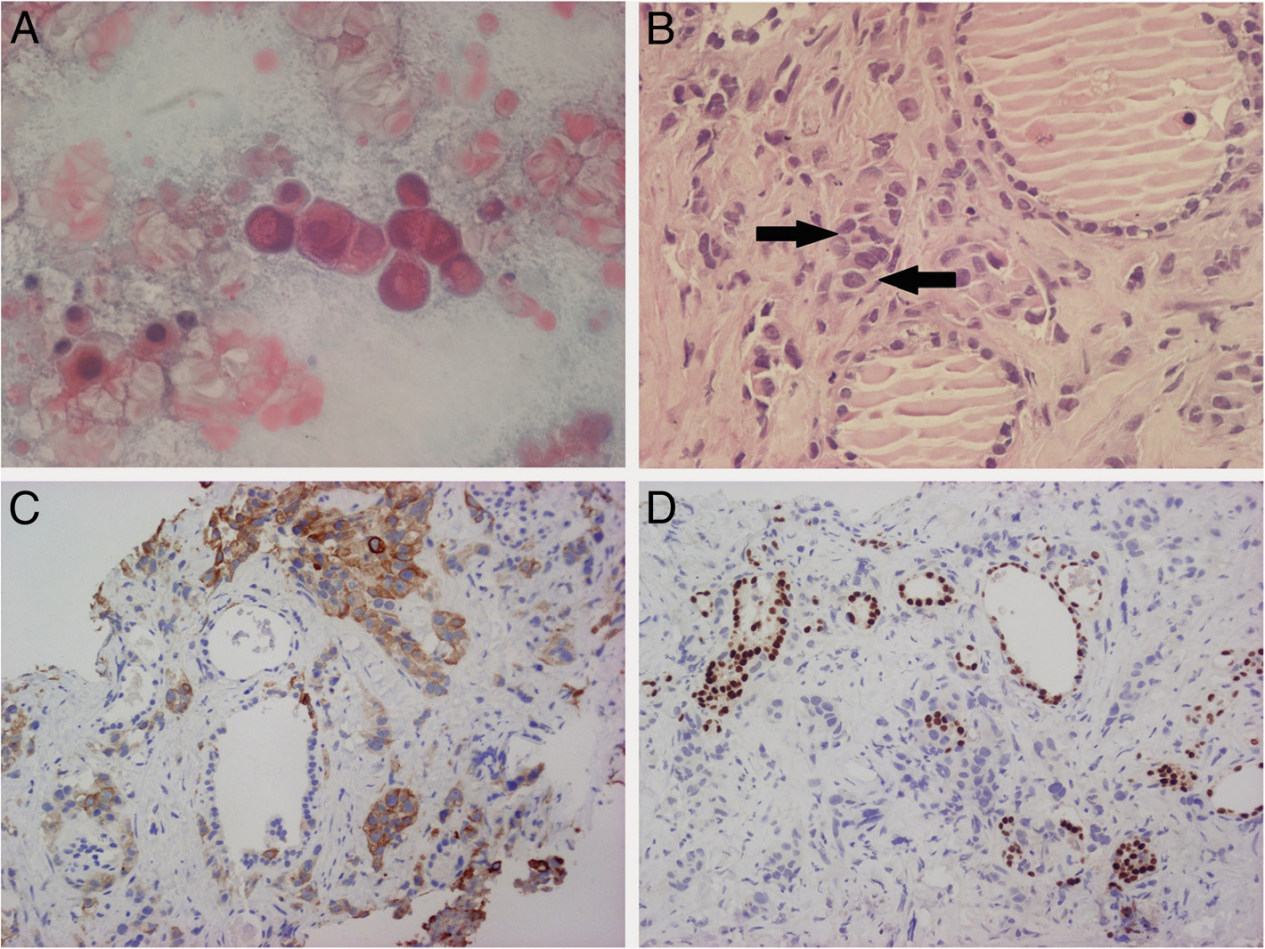
**Cytological and histological aspects of the nodule. (A)** Thyroid fine needle aspiration (Papanicolaou, magnitude 600 X). A high power field showing a cluster of neoplastic cells in a necrotic background. **(B)** Thyroid core biopsy (hematoxilin and eosin, magnitude 400 X): thyroid tissue infiltrated by poorly differentiated adenocarcinoma (arrows). **(C)** Thyroid core biopsy (CK19, magnitude 200 X): Strong cytoplasmic positivity in neoplastic cells. **(D)** Thyroid core biopsy (TTF-1, magnitude 200 X): nuclear positivity in follicular cells.

As an outpatient he underwent a neck ultrasonography (US) in another center which identified a big hypoechoic lesion in the right lobe with ill-defined margins. Cytological analysis of the lesion was defined as malignancy (Figure 1A) according to the Bethesda System for reporting thyroid cytopathology [5] and the patient was referred for possible thyroidectomy. Thyroid function tests showed the following results: thyrotropin level 5.5 μU/ml (normal 0.46-4.68), free triiodothyronine 3.12 pg/ml (normal 2.75-5.26), and free thyroxine 1.07 ng/dl, (normal 0.77-2.19). Antibodies against thyroperoxidase and against thyroglobulin were negative, and serum calcitonin was normal (8 pg/ml, normal < 18).

**Figure 2:**
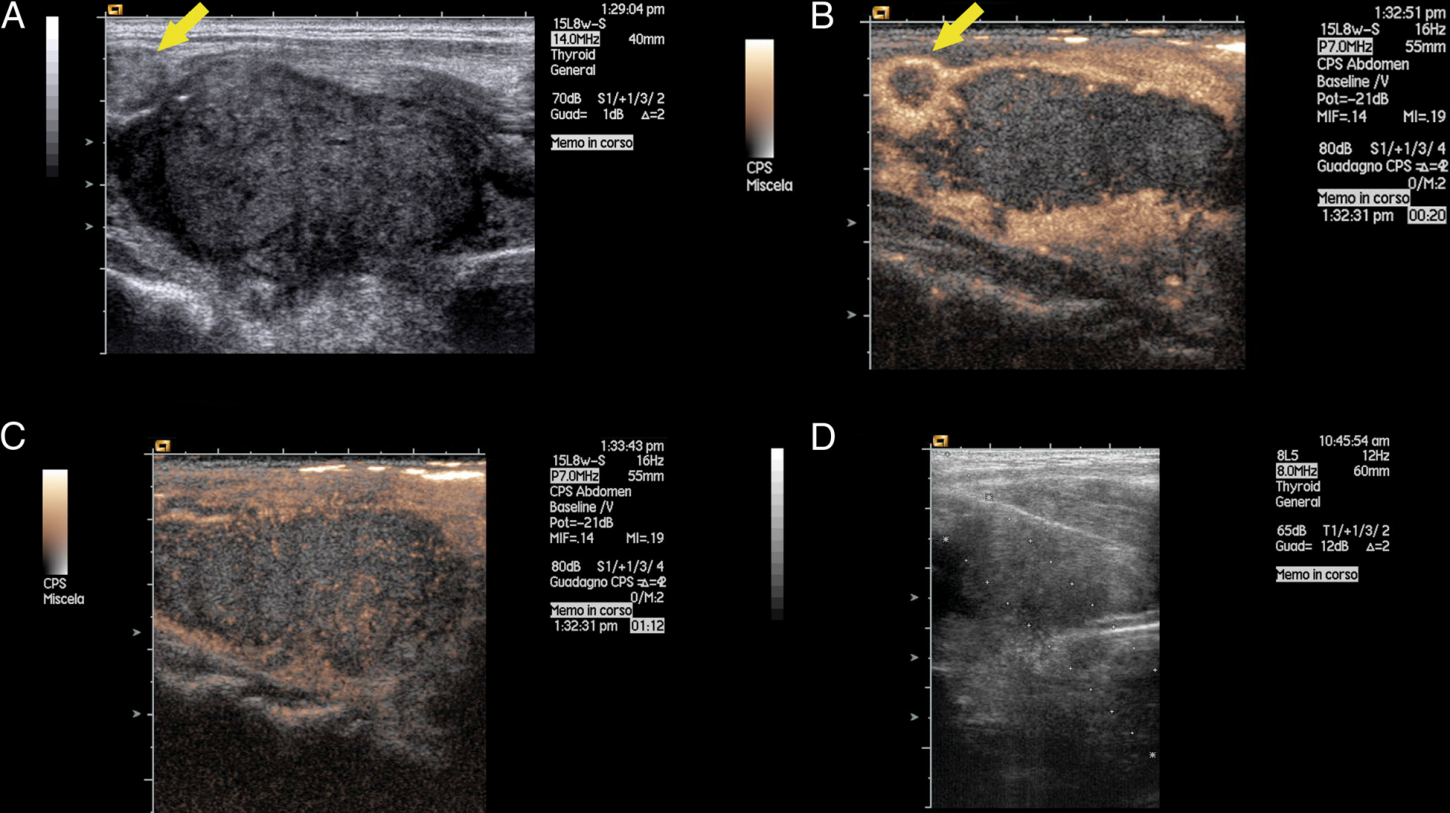
**Thyroid ultrasound with contrast enhancement and biopsy.** Longitudinal view of the right lobe of the thyroid gland performed with linear probe (7.5-14 Mhz) in conventional ultrasound **(panel A, D)** and after contrast enhancement ultrasound **(panel B,C)**. Panel A shows a hypoechoic lesion of the right lobe of the thyroid gland. The yellow arrow indicates a metastatic lymph node located close to the upper pole of the right lobe. Panel B shows the hypovascular aspect of the nodule and lymph node after contrast injection. The enhancement is located only in the peripheral part both of nodule and lymph node as well. Panel C shows the enhancement in the venous phase which is still located only in the peripheral part of the nodule, except for the presence of small tiny vessels located also in the central part. Panel D show the path of the needle inserted inside of the nodule.

Subsequently, he referred to our ward to perform further analysis. Neck US was repeated using a high frequency linear probe (15 L8W, 7.5-14 MHz, Acuson Sequoia 512, Siemens, Mountain View, California, USA). A thyroid hypoechoic mass of 30 mm in the maximum longitudinal size was detected in the right lobe of the gland, associated with round and enlarged lymph nodes in laterocervical part of the neck, especially on the right side. After conventional US, a contrast enhancement US (CPS, Siemens, Mountain View, California; Sonovue, Bracco, Italy) focused to the thyroid gland and lymph node was performed (Figure 2). Nodule and lymph node showed a similar pattern. In the central part, they exhibited a hypovascular aspect in all phases of contrast US (typical for malignant lesions), except for the presence of small vessels. The enhancement was located only in the peripheral part of both nodule and lymph node.

An US-guided core biopsy (Selectcore Inrad, 20 gauge, 10 cm in length) of the nodule was performed.

On microscopic examination, histological sections revealed a pseudoglandular proliferation of atypical epithelial cells embedded in a fibrous stroma with atrophic thyroid follicles (Figure 1B). Furthermore, on immunohistochemical findings, thyrocytes showed immunoreactivity for thyroid transcription factor-1 (TTF-1) in spite of negativity in the neoplastic cells (Figure 1D). All these features were consistent with the diagnosis of metastatic pancreatic adenocarcinoma. Therefore, we measured tumor markers which revealed elevated carcinoembryonic antigen (15.98 ng/mL, normal <5), cancer antigen 19–9 (8731.52 U/ml, normal <37), and slightly elevated cancer antigen 125 (240.10 U/ml, normal <35). Additional laboratory blood tests were consistent with biliary obstruction and liver damage.

**Figure 3:**
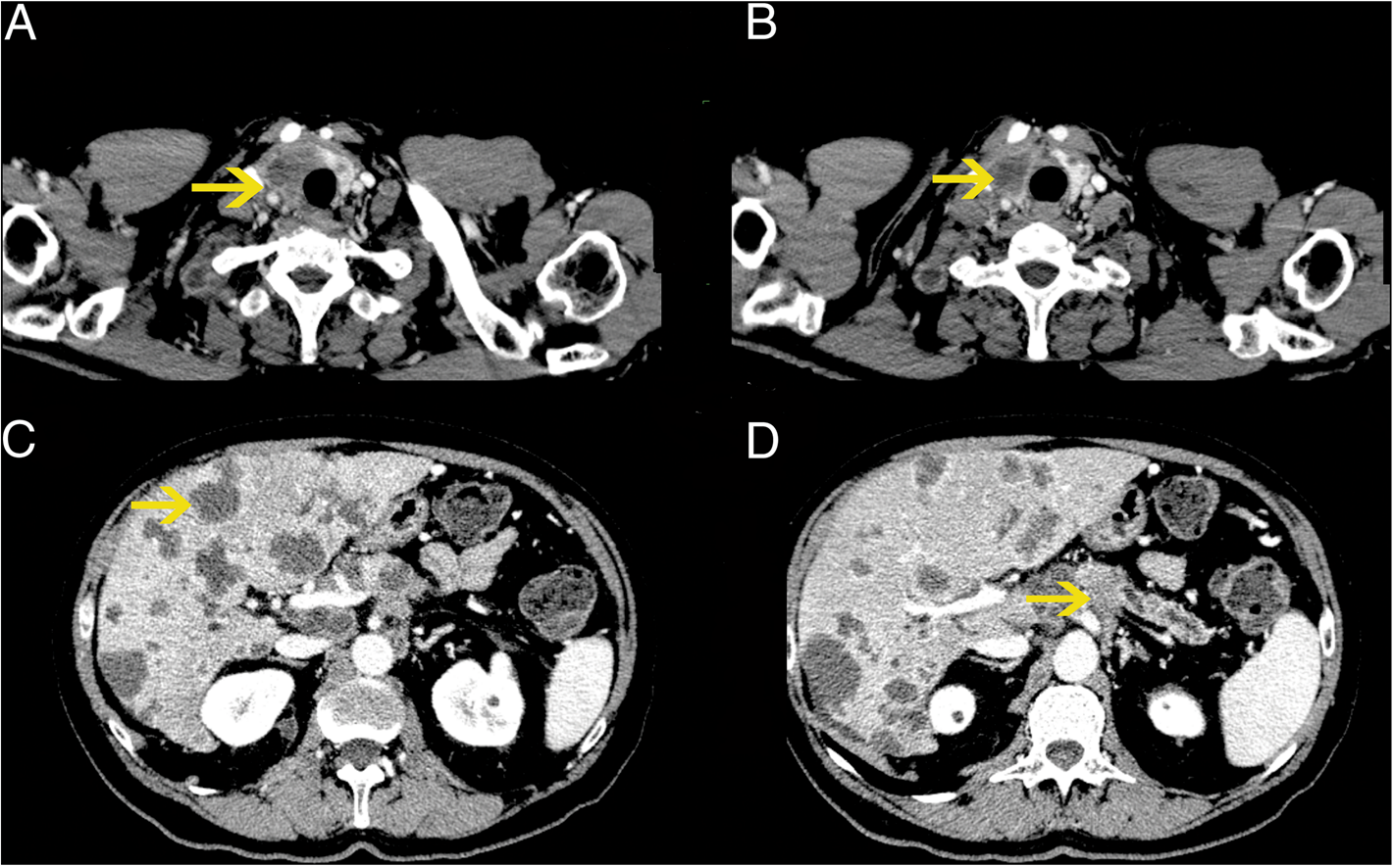
**CT scan of the body.** In panel **A** and **B** the yellow arrows indicate the big mass of the right lobe of the thyroid gland. In panel **C** the yellow arrow indicates one of the multiple secondary liver lesions. In panel **D** the yellow arrow indicates the pancreatic cancer located in the body with dilatation of the pancreatic duct.

A contrast CT scan demonstrated an enlargement of the pancreatic body with a 22 mm hypodense lesion with ill-defined margins, dilatation of the pancreatic duct, diffuse retroperitoneal, paraaortic and cervical lymphadenopathy and secondary lesions in the liver (1–4.2 cm) (Figure 3). A needle biopsy of the largest hepatic lesion revealed hepatic tissue infiltrated by poorly differentiated adenocarcinoma which was similar to the neoplastic tissue found in the right lobe of the thyroid.

The patient referred to the oncology unit for palliative treatment, and died two months later.

## Discussion

The thyroid gland is believed to be a rare site of metastatic disease. It has been hypothesized that the fast arterial flow through the thyroid and the high oxygen saturation and iodine content within the thyroid might inhibit the growth of metastatic malignant cells [3]. NTMs seem to occur more frequently in glands that were also abnormal. These abnormalities included goiter, thyroiditis, benign and malignant primary neoplasm. In particular, malignant metastases were most commonly found concomitantly with goiters and follicular thyroid adenomas [6]. These data support the hypothesis that the abnormal blood supply caused by concomitant thyroid abnormalities might result in decreased oxygen and iodine content.

The reported prevalence of metastasis to the thyroid is variable, since the prevalence of thyroid metastasis from NTMs ranges from 1.9% to 24% in autopsy studies in patients with a known history of neoplasm. This wide range may be mainly explained by different population studied (few cases in most of the studies) and by the predominance of the type of primary cancer included in the series (patients with renal cancer had an increased prevalence of metastasis to thyroid gland).

However, a very recent review of the literature clearly suggests that clinically significant metastases of NTMs to the thyroid are not rare. In particular, the most common NTMs that metastasize to the gland are renal cell, colorectal, lung, breast carcinoma and sarcoma [2,6]. Thus, metastasis to the thyroid should be considered when a thyroid lesion is detected in the follow-up of patients with a history of cancer.

According a MEDLINE search, there are only three reported cases of thyroid metastasis from pancreatic malignant tumour.

The first was reported by Eriksson et al. [7] in a 54 year-old men with disseminated pancreatic carcinoma. Interestingly, this patient had a transient thyrotoxicosis due to a destructive thyroiditis caused by the massive invasion of the organ by tumour cells. Percutaneous biopsy of the gland excluded the presence of thyroiditis and demonstrated the presence of carcinoma. Autopsy showed an adenocarcinoma of the pancreatic tail with widespread metastasis. In particular, in the areas involved by the tumour, invasion and disruption of thyroid follicles by tumour cells were frequent.

The second patient was reported by Hsiao et al. [8] in a 45-year-old male with a previously diagnosed intraductal papillary-mucinous carcinoma of the pancreas. Adenocarcinoma metastatic to the thyroid was proven pathologically only after thyroidectomy, as cytodiagnosis of the thyroid aspirate did not proven the primary site of the lesion. The patient died one week after the surgery.

The third case has been reported by Kelly et al. [9] in a 38-year-old man with obstructive jaundice and epigastric pain who underwent a pancreatic-duodenectomy and, eight weeks later, a total thyroidectomy. The histology of the thyroid was similar to that of the pancreatic specimen.

Metastatic lesions in the thyroid gland could range from overt hypothyroidism with myxedema (3) to hyperthyroidism/thyrotoxicosis (7), although most of patients did not show any thyroid dysfunction. The prognosis essentially refers to the primary site of the cancer, and the overall survival time is not significantly different in cancer patients with or without thyroid metastasis [10].

Fine needle aspiration (FNA) is the most common and useful investigation in the diagnosis of thyroid pathology. The technique is also useful in the diagnosis of malignant metastasis to the thyroid. However, FNA may not yield a definitive diagnosis in all cases, particularly when the metastatic cells are poorly differentiated. Therefore histology and immunochemistry is usually necessary in order to differentiate primary thyroid carcinomas from metastatic neoplasm. Percutaneous core biopsy has been shown to be superior to the FNA in patients with prior non diagnostic FNA of thyroid nodules [11]. In our patient microscopic examination of the specimen obtained by core biopsy revealed the presence of a poorly differentiated carcinoma with a strong cytoplasmatic positivity for Cytokeratin 19 [12], and negative staining for thyroglobulin and TTF-1 [13], thus favouring the diagnosis of metastatic tumour over a primary thyroid cancer. The association of this immunohistochemical finding and clinical and biochemical data clearly indicated that the thyroid lesion was a metastatic pancreatic carcinoma.

## Conclusions

Our case represents the fourth case reported in the literature of pancreatic adenocarcinoma metastatic to the thyroid, and the first in whom the thyroid metastasis was the initial clinical presentation of the pancreatic disease. The core biopsy of the thyroid lesion, providing more tissue (with retained cellular architecture) compared to the FNA, allowed the identification of the primary neoplasm. Moreover, we describe for the first time the behavior of the ultrasound contrast agent in a thyroid lesion, secondary to a metastatic pancreatic adenocarcinoma.

Metastases of NTM to thyroid gland are uncommon and their identification could be challenging. We suggest that the use of ultrasound-guided core biopsy could help the physician in the diagnosis.

### Consent

Written informed consent was obtained from the patient for publication of this Case report and accompanying images. A copy of the written consent is available for review by the Editor of this journal.

## Abbreviations

NTMs: Nonthyroid malignancies; US: Ultrasonography; TTF-1: Thyroid transcription factor-1; FNA: Fine needle aspiration.

## Competing interest

The authors declare that they have no competing interests.

## Authors’ contributions

APD: wrote the manuscript, GPV: performed contrast enhanced ultrasonography and core-biopsy, AM: performed immunohistochemical staining of the lesion, DU: data collection, GD: reviewed and edited manuscript, GF: reviewed and edited manuscript. All authors read and approved the final manuscript.

## Pre-publication history

The pre-publication history for this paper can be accessed here:

http://www.biomedcentral.com/1472-6823/14/6/prepub
